# PP64 Impact Of The Cost-Effectiveness Threshold On Drug Funding Recommendations: The Case Of Rare Diseases In Brazil

**DOI:** 10.1017/S0266462324002356

**Published:** 2025-01-07

**Authors:** Eduardo Oliveira, Luciana Xavier, Priscila Louly, Clementina Prado, Luciene Bonan

## Abstract

**Introduction:**

In 2022, the Brazilian public health system has adopted an explicit cost-effectiveness threshold of USD24,232.91 per quality-adjusted life years (QALY) for evaluating technologies intended for rare diseases treatment. Although regarded as a strategy for increasing efficiency, the National Committee for Health Technology Incorporation (Conitec) has recommended that the threshold should not be used as a knockout parameter.

**Methods:**

A retrospective analysis of Conitec’s recommendations regarding technologies for rare diseases issued between January and October 2023 was conducted. The following data were extracted from Conitec’s reports: (i) disease (rare or ultra-rare); (ii) health technology evaluated; (iii) scientific evidence regarding efficacy, effectiveness, and safety of the technology; (iv) incremental cost-effectiveness ratio (ICER); (v) recommendation issued; and (vi) rationale for recommendation. Identified technologies will be divided into two groups according to the recommendation issued (positive or negative). The rationale for each recommendation, along with other information, will be reviewed to look for explicit threshold utilization and its impact on decision-making.

**Results:**

Twelve technologies for nine rare diseases evaluated in 2023 were retrieved and eleven were included in the analysis. Six drugs have received a positive recommendation for funding. The ICERs estimated varied between ‒USD5,278,362.06 for emicizumab for hemophilia A and USD116,407.47 for elexacaftor/tezacaftor/ivacaftor for cystic fibrosis. Two of them have received a positive recommendation despite their associated ICER exceeding the explicit threshold. All negative recommendations were associated with ICERs higher than the threshold, which varied between USD52,456.32 and USD847,942.97. For two, the threshold was specifically mentioned as the rationale for the negative.

**Conclusions:**

After the adoption of an explicit cost-effectiveness threshold in 2023, Conitec has issued two positive funding recommendations for rare diseases technologies despite their associated ICER exceeding the threshold: agalsidase alpha for Fabry disease and elexacaftor/tezacaftor/ivacaftor for cystic fibrosis. For both recommendations, the drug’s favorable impact on the natural history of the disease was considered decisive.

